# Maternal Intake of Polyunsaturated Fatty Acids in Autism Spectrum Etiology and Its Relation to the Gut Microbiota: What Do We Know?

**DOI:** 10.3390/nu15071551

**Published:** 2023-03-23

**Authors:** Elisana Lima Rodrigues, Priscila Silva Figueiredo, Gabriela Marcelino, Rita de Cássia Avellaneda Guimarães, Arnildo Pott, Lidiani Figueiredo Santana, Priscila Aiko Hiane, Valter Aragão do Nascimento, Danielle Bogo, Karine de Cássia Freitas

**Affiliations:** 1 Graduate Program in Health and Development in the Central-West Region of Brazil, Federal University of Mato Grosso do Sul-UFMS, Campo Grande 79079-900, MS, Brazil; 2 Institute of Biosciences, Federal University of Mato Grosso do Sul-UFMS, Campo Grande 79079-900, MS, Brazil

**Keywords:** infant neurodevelopmental, linoleic acid, linolenic acid, maternal food quality, maternal gut microbiota, offspring microbiota

## Abstract

Maternal food habits and gut microbiota composition have potential effects on fetal neurodevelopment, impacting Autism Spectrum Disorder (ASD). Our research aims to outline the relationship that ingestion of polyunsaturated fatty acids (PUFAs) and the composition of maternal gut microbiota have with the possible development of ASD in offspring. We suggest that genetic factors could be related to the different conversions between unsaturated fatty acids according to sex and, mainly, the impact of the pregnancy diet on the higher or lower risk of neurological impairments. The proportion of the phyla Firmicutes/Bacteroidetes is high with an increased consumption of linoleic acid (LA, n-6 PUFA), which is associated with maternal intestinal dysbiosis and consequently starts the inflammatory process, harming myelinization. In contrast, the consumption of α-linolenic acid (ALA, n-3 PUFA) tends to re-establish the balance of the maternal microbiota with anti-inflammatory action. Moreover, human observational studies showed a strong correlation between the consumption of n-3 PUFA, mainly above 340 g of fish per week, with beneficial effects on infant neurodevelopment. Therefore, we suggest that the proper intake of foods rich in n-3 PUFAs and their supplementation during pregnancy until lactation has an impact on reducing the development of ASD. Controlled studies with n-3 PUFA supplementation are still necessary to verify the ideal dose and the best form of administration.

## 1. Introduction

Autism Spectrum Disorder (ASD) is considered to be a neurodevelopmental disturbance and its characteristics are present from childhood; they depend on the individual and environmental particularities. ASD symptoms are mainly described by interaction and social communication difficulties, repetitive behavior patterns, and sensory integration disorder (SID), which cause challenging behaviors [1]. Concerning SID, one study reports that children with ASD, compared with children with typical development, show an unbalance with reduced levels of gamma-aminobutyric acid (GABA), a leading inhibitory neurotransmitter with a crucial role in SID [2]. Therefore, most children with ASD have impaired sensory input, such as sound, touch, body movement/position, vision, taste, and smell, which result in difficulties in behaviors of externalization and internalization, emotional and attention regulation, executive function, and functional activities of daily life, including social activities [3,4], which can generate behavioral problems, such as verbal anger (cry, argue, scream, and yell) and physical anger outbreaks (kick, hit, and throw toy), besides self-harm violence that affects their safety [5].

Due to the generalized impact on daily life abilities and occupational performance, sensory-based intervention (SBI) options are recommended to deal with the difficulties. An example of SBI was presented in the experimental single-blind study of Afif et al. [6], who utilized two models of an autism hug machine portable seat (AHMPS) to determine the effect of its short-term use in improving behavioral and neurobiological stress in 20 children with ASD aged between 7 and 13 years. The infant behavioral data, evaluated through the classification scale of Conners (CPRS-48), revealed a significant improvement in behavior problems, psychosomatic problems, impulsive-hyperactive behaviors, and anxiety.

In the USA, in 2016, the recorded prevalence of children diagnosed with ASD was one in every 40 [7]. Although studies are becoming more detailed, with populational samples and more accurate trials, the data concerning the prevalence of ASD have been questioned as there is no standardization of methodologies for research and diagnosis, thus increasing the difference in prevalence among studies [8].

The etiopathogeny of ASD is still not defined; however, it is understood as a multifactorial pathogenesis, dependent on an implicit biological vulnerability, conditioned by the different degrees of exogenous stressors experienced during the period of intrauterine and post-birth brain development [9]. Studies indicate that prematurity [10,11] and low birth weight [12] are risk factors for the later development of comorbidities of neurodevelopment, including motor deficiencies, socio-behavioral deficits, and ASD [13].

Nevertheless, the lifestyle of the mother is the most relevant factor for the risk of development of ASD. The maternal–fetal physiology is influenced by multiple risk factors between pregnancy and the first days after birth, such as stress, the use of medications, breastfeeding, and diet. Moreover, the alteration of the maternal gut microbiota is crucial as a risk factor, since it is linked to the digestion, physiology, and gastrointestinal immunity of the children through the microbiota–intestine–brain axis [14]. Furthermore, it is worth highlighting that the intestine of the newborn can be influenced by the maternal microbiota through the vertical transference of microorganisms to the baby during vaginal delivery and breastfeeding [15].

Maternal obesity during pregnancy and the eventual imbalance of the microbiota could correlate with the development of neurological disorders [16,17,18,19]. The main mechanisms whereby maternal obesity can affect infant neurodevelopment are related to the concentrations of maternal pro-inflammatory cytokines since they can cross the hemato-placental barrier and interact with fetal neurodevelopment, leading to factors that include neuroinflammation, increased oxidative stress, dysregulated insulin signaling, glucose and leptin, serotonin-synergistic and dysregulated dopaminergic signaling, and synaptic plasticity disorders [18].

Maternal nutrition can also be a crucial risk factor for ASD since poor food variety and nutrient deficiencies are strongly associated with neurodevelopmental disorders in children. For example, unbalanced levels of essential fatty acids, especially polyunsaturated fatty acids (PUFAs), are observed in patients with ASD and other neurological development disorders, such as attention deficit and hyperactivity disorder (ADHD) and schizophrenia. Curiously, PUFAs, specifically the n-3 PUFAs, are potent immunomodulators that exert anti-inflammatory properties in the brain, regulating microglia activity [20]. Preliminary research evidence indicates that the deficient maternal ingestion of omega-3 and linoleic fatty acids can increase the risk of ASD in the offspring [21].

This review aims to document the existing evidence in the literature on the impact of the maternal food ingestion of PUFAs on the development of ASD, focusing primarily on the relationship between dietetic lipids and alterations in the composition of the maternal microbiota intestinal once few studies investigated that correlation. Developing a broader comprehension of the mechanisms of the intestine–brain axis and the influence of dietetic fatty acids can contribute to defining guidelines for preventing ASD through nutritional interventions. The aims of this study are to investigate maternal nutrition correlated with lipidic consumption during pregnancy and its relationship with the origin of Autism Spectrum Disorder, also considering maternal gut microbiota and other neurological development disorders.

## 2. Methodology

Authors searched PubMed of the National Library of Medicine, MDPI magazine special editions, and Google Scholar. Databases were extensively searched for all original and review articles, as well as book chapters and published abstracts using keywords (single or in combination): Autism, autism spectrum disorder, the prevalence of autism spectrum disorder, maternal intake, offspring, dietary omega-3, polyunsaturated fatty acids intake, PUFAs intake, neuroinflammation, behavioral symptoms, nutritional supplementation, risk factors for autism, maternal lifestyle, pregnancy and fatty acid intake, maternal microbiota, pregnant gut microbiota, and DHA intake published in English until December 2022. Additional articles in theoretical references of reviewed articles were also searched. In summary, the most relevant articles were included after evaluation.

## 3. Understanding Autism Spectrum Disorder: General Concepts

The disorders classified within the context of ASD are generally manifested in the first stage of infancy, characterized by deficits in development, with impairments across life in personal, social, academic, or professional functioning [1]. The developmental deficits vary from particular limitations in learning or the control of executive functions to global losses in social or intellectual abilities, often with occurrence of the association of more than one disorder during neurodevelopment—for example, the presence of attention deficit and hyperactivity disorder in children with ASD [1]. Moreover, ordinarily, patients can present some level of intellectual deficiency [22] and a convulsive disorder in severe cases [23]. In addition, some individuals can show anxiety, learning delays, sensorial sensibility, and motor deficits [1].

The behavioral characteristics of ASD initially become evident in the first stage of infancy (from birth to 5 years of age), with some cases presenting a lack of interest in social interactions in the first year of life [1]. Such characteristics can also begin late, named regressive autism or late start autism, which describes a subgroup of patients with initially normal development but with a gradual loss of abilities in communication or social interaction [24].

Some children with ASD present plateaus or regression in development, with a gradual or relatively fast deterioration in social behaviors or language use. Such losses are rare in other neurodevelopmental disorders; for example, intellectual development disorder can be a helpful indicative sign of ASD [1]. Another reported behavior is fussy eating habits, which can cause deficiencies in vitamins, minerals, and fatty acids [25]. In a metanalysis, it was observed that children within the age range of 4–13 years and with ASD have significantly lower ingestion of omega-3, protein, calcium, phosphorous, selenium, vitamin D, thiamin, riboflavin, and vitamin B12 and higher ingestion of polyunsaturated fats (PUFAs), simple carbohydrates, and vitamin E than children with typical development [26].

Epidemiological studies have shown a four to five-times higher prevalence of ASD in boys than in girls. In Asia, Europe, and North America, it is estimated at 1% [27,28]; in the United States, the prevalence of ASD among 8-year-olds was 1 in 59 in 2014 and 1 in 54 in 2016 [29], and the prevalence in children and adolescents was reported at 2.5% in 2014–2016 [30,31]. In Italy, the prevalence of ASD among children aged 7 to 9 years was 1.15% [32], with a continuous trend of increasing diagnoses of autism, arousing the interest of the scientific community [33,34,35]. ASD is only diagnosed when the deficits in social communication are followed by excessively repetitive behaviors, restrictive interests, and insistence on the same routines [1].

Genetic and environmental factors are implied in the etiology of ASD [36,37]. It generally affects more males, with a mean proportion of 4:1 of men to women [29], possibly due to specific genetic differences [38]. Although it cannot be uniquely attributed to this issue, and there is no definitive explanation, a point to be noted is that there are differences in the conversion rate of essential fatty acids according to gender. Women have a higher capacity for converting alpha-linolenic acid (ALA) into eicosapentaenoic acid (EPA) and docosahexaenoic acid (DHA) [39,40,41]. Moreover, it is worth pointing out that DHA is an essential molecule for intracellular signaling [42], involved in the regulation of genes [43], correlating with genetic defects associated with chromosome X, for example, fragile X syndrome [44].

Despite not being well established, research points to a possible link between ASD and the gut microbiota [45], which is correlated with the type of childbirth, breastfeeding time, gestational stress, use of antibiotics, and maternal diet [46].

## 4. The Role of PUFA Intake during Pregnancy and Its Risk for the Development of Autistic Traits

Briefly, as previously mentioned, feeding plays a vital role during pregnancy in fetal development and metabolism, mainly influenced by nutrient quality in fetal programming. There is evidence that nutritional factors, including energy, fatty acids, proteins, and micronutrients such as folate, affect various aspects of metabolic programming [47].

Polyunsaturated fatty acids (PUFAs) are characterized by the presence of two or more double bonds among the carbon atoms in their structure, which are classified as n-6 PUFAs and n-3 PUFAs. PUFAs are ordered in long-chain PUFAs (LC-PUFAs, fatty acids ≤ C20), such as linoleic (LA, n-6 18:2) and α-linolenic acids (ALA, n-3 18:3); acting via physiological mechanisms and reactions, these are precursors of very long-chain fatty acids (VLCFAs ≥ C22). Endogenously, LA is converted into arachidonic acid (AA, n-6 C20:4), whereas ALA is converted to EAP (n-3 C20:5) and DHA (n-3 C22:6) [48]. The n-3 series PUFAs, mainly EPA and DHA, are considered bioactive compounds due to their relevant functional and structural roles in the cell membrane. In addition, PUFAs act by signaling a series of processes, such as prostaglandin synthesis, associated with critical biological activities during pregnancy, such as vasodilation, placental blood flow, cervical ripening, and labor initiation [49]. Both n-3 PUFAs and n-6 PUFAs are essential nutrients due to the lack of specific enzymes, such as desaturases, that mammals cannot synthesize [50]. Therefore, the consumption via food sources of two essential fatty acids of 18 carbons, LA and ALA, is necessary to generate n-6 PUFAs and n-3 PUFAs biologically active [51].

The n-3 and n-6 PUFAs are considered essential fatty acid modulators of inflammatory cascades, which maintain the fluid integrity of cellular membranes [52], associated with neurological development and the fetal immune system [53]. Based on this leading influence on fetal programming, Table 1 shows the effects of PUFA consumption during pregnancy and its association with offspring autistic traits.

A cohort study evaluated whether the maternal plasmatic PUFA concentrations and the proportions of n-3 and n-6 during pregnancy affect the risk of autistic traits in the offspring at 6 years of age. No association was found between n-3 maternal levels and autism traits in the child, defined in the study by its intelligence quotient (IQ), which takes into account prenatal PUFA status associated with the child’s general neurodevelopment and other scores of global cognitive ability [54]. In contrast, an association occurred with higher plasmatic levels of n-6 PUFA. Moreover, the study showed that a lower pre-natal proportion of n-3:n-6 is associated with more infant autistic traits, widely explained by a higher level of n-6. Thus, the results suggest a biological path between the maternal ingestion of fatty acids during pregnancy and autistic traits in the offspring [55]. Another prospective cohort investigation emphasizes that the maternal ingestion of higher levels of omega-3 during the second half of pregnancy reduced by 40% the risk of child ASD [56].

**Table 1:** Associations of long-chain polyunsaturated fatty acid (LCPUFA) intake during pregnancy with child autistic traits.

Study Design/Kind of Study	Sample	Diet	Child Autistic Traits
− Maternal polyunsaturated fatty acid status during pregnancy − Generation R cohort [55]	3802 mothers	Low intake or concentrations of ω-3	No associations of individual n-3 PUFAs with child autistic traits
		Higher total ω-6 levels (linoleic acid only)	More child autistic traits
− Markers of Autism Risk in Babies-Learning Early Signs − (MARBLES) study—Prospective cohort study − Prospective cohort study [56]	258 mother-child pairs	Higher consumption of total n-3 in second half of pregnancy	40% lower risk of having children with Autism Spectrum Disorder
− Japan Environment and Children’s Study (JECS) − Prospective cohort study [57]	92,011 BAP mothers	Pregnant women with higher BAP level presented lower consumption of vegetables and fish	A potential risk factor for children’s food choices, mental and physical development
− Spanish Childhood and Environment − Project − Multicenter birth cohort study [58]	1892 and 1589 mother-child pairs at the ages of 14 months and 5 years, respectively	Consumption of seafood by pregnant women over 454 g/week	A consistent reduction in Autism Spectrum Disorder traits
− Avon Longitudinal Study of Parents and Children (ALSPAC) − Observational study [59]	11,875 pregnant women	Maternal consumption of seafood over 340 g per week	Beneficial for the children’s neurodevelopment (considering verbal IQ and communication skills)

ASD: Autism Spectrum Disorder; BAP: broad autism phenotype; ALA: α-linolenic acid; LA: linoleic acid; PUFA: polyunsaturated fatty acid.

The Japan Environment and Children’s Study investigated the associated dietary intake of several micronutrients, such as vitamins C and D, folate, and n-3 PUFAs, in pregnancy with the broad autism phenotype (BAP), and results presented behavioral and cognitive disorders similar to autistic aspects, but lower than threshold levels. Mothers with high levels of BAP had high food selectivity, low vegetable and fish consumption and, consequently, consumed few n-3 PUFAs. This type of maternal food preference was reflected in their children until five years of age [57]. Such observations suggested an association among the potential low consumption of vitamin D and n-3 PUFAs with impairing dietary effects on child brain development and function [60,61], which could suggest a decisive intervention in the treatment of pregnant women with BAP status [57].

Positive effects of n-3 PUFA consumption were reported for pregnant women that ate ≥ 450 g of fish and seafood compared with the recommended 340 g per week; from the last period of the diet, the low incidence of neurophysiologic dysfunctions in their children of 14 months and five years old was notable [58]. Such an association proved that neurodevelopment in gestation is marked by intense formation, differentiation, and neural migration activities [62], demonstrating the crucial role of the adequate consumption of n-3 PUFAs during pregnancy.

Likewise, the Avon Longitudinal Study of Parents and Children (ALSPAC) showed that the maternal consumption of n-3 PUFAs in seafood > 340 g per week was associated with child protection and sound development, whereas < 340 g per week demonstrated a greater risk to children, resulting in prosocial behavior and motor, communication, and social development [59]. However, another critical point is the high mercury levels in the recommended diets due to their hazardous effects on children and on pregnant and nursing women and those who wish to become pregnant, being advised to eat quality fish for two meals per week and following the recommendations of the Food and Agriculture Organization of the United Nations and Environmental Protection Agency (FAO/EPA). Nevertheless, the benefit of frequent fish consumption is greater than the harm posed by other heavy metals [63].

Therefore, maternal nutrition quality during gestation and/or breastfeeding is strongly associated with the fetus, neonate, and older stages of life in terms of programming structural and functional physiology regulation and preventing several diseases throughout life [64]. Further studies of the correlation of the amount of n-3 and n-6/n-3 PUFA in the diet in pregnancy, lactation, and in women who wish to become pregnant are needed for health improvement, including within the context of ASD and associated diseases for children and other subsequent stages of life.

## 5. Gut Microbiota of Pregnant Women: A Link with the Child’s Autism Spectrum Disorder?

During pregnancy, the maternal microbiota modulates the function of the offspring’s microglia, which play a central role in brain development and its plasticity [65,66,67]. Evidence indicates that the maternal microbiota can lead to the development and the microglial function of the offspring, which depends on the integrity of maternal gut–brain crosstalk [68]. Besides, pre-natal intestinal microbiota alterations in the first years of life can determine the state of severe immunologic alterations, including the production of inflammatory cytokines and persistent activation of microglia cells. It is known that unhealthy food patterns, such as the consumption of saturated fats, are directly linked to low-degree systemic inflammation, obesity, and pro-inflammatory immune response [69,70]. Furthermore, data reveal that the neuroinflammatory signaling driven by microglia is a causal link between the excessive consumption of a high-fat diet (HFD) and hypothalamic gliosis, resulting in a key element in the brain inflammation induced by HFD and energetic unbalance [71]. If unhealthy food patterns are potent determinants of the alteration of the host microbiota and the dysbiosis generates microglial hyperactivity, the consumption of selected dietetic lipids can contribute considerably to controlling the activation of microglia and brain inflammation, thus, reduce the risk for neuropsychiatric diseases [72].

**Figure 1:**
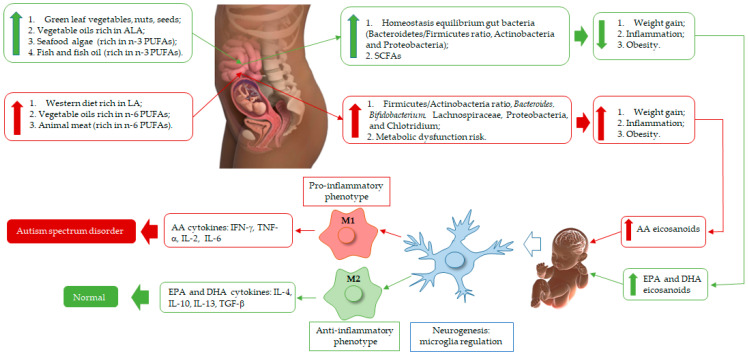
Schematic representation of n-3 and n-6 polyunsaturated fatty acids (PUFAs) rich diet of pregnant woman, fetus, and neonate brain development. EPA and DHA produce eicosanoids (resolvins, protectins) with anti-inflammatory effects and modulate the membrane patterns of lymphocytes, macrophages, and neutrophils, decreasing AA content. These biosynthesis events are correlated with macrophage 2 (M2) action and equilibrium homeostasis conditions in fetus and baby brain health during its development, and their eicosanoids inhibit or slow down arachidonic acid (AA), which acts as an anti-inflammatory agent for neurogenesis health improvement. On the other hand, vegetable oils and animal meat (poultry, beef, pig) are rich in linoleic acid (LA). Thus, in the body, LA is bio-converted to AA, which is correlated with eicosanoid production (prostaglandins (PGs), leukotrienes (LTs), and lipoxins (LXs)), linked to molecule signaling in the oxidative pathway of AA and macrophage 1 (M1) recruitment, correlated with pro-inflammatory processes, which damage the microglia, affecting normal brain regulation and development, which then may lead to Autism Spectrum Disorder. These regulation processes begin when a woman is pregnant (fetus) until the breastfeeding of her baby. Thus, infant neurogenesis health is governed by the type of PUFA and eicosanoid components accumulated in the brain, linked to maternal diet.

The intestine–brain axis corresponds to signaling between the enteric and central nervous systems, and the gut microbiota has an outstanding role in this scenario [73]. Dysregulation in this axis has been related to the development of autism across its diversity of symptoms [20], as represented in Figure 1.

Gut dysbiosis is commonly associated with increased intestinal permeability. Thereby, intestinal bacteria can cross this barrier and reach the mesenteric lymphoid tissue, which will cause the increased production and liberation of inflammatory cytokines such as Interleukin 1 (IL-1) and Interleukin 6 (IL-6). These cytokines can cross the hematoencephalic barrier and activate the hypothalamic–hypophysis–adrenal axis (HPA), leading to the liberation of cortisol, which further activates the stress system. Since the central nervous system and the gastrointestinal tract are intimately related, stress can affect the presence and abundance of some gut microorganisms, i.e., results in the alteration of the structure of the microbiome induced by stress. Such factors could cause brain alterations characteristic of autism, besides the clinical signs [74].

During pregnancy, through the placenta and during lactation, the intestinal microbiota of the neonate is influenced by different factors, such as the maternal diet. This can be reflected in its health and directly impact its physiology, cognition, and other aspects [75]. The qualitative change in the diet concerning the lipid composition is reflected directly in the gut microbiota. A study with aged C57BL/6 mice observed that adding 20% (*w*/*w*) of corn oil and 20% (*w*/*w*) of rapeseed oil, rich in n-6 PUFAs, to the diet significantly changed the microbiota composition, raising the ratio between the phyla Firmicutes and Bacteroidetes [76]. Alterations in the ratio between these phyla can promote intestinal dysbiosis associated with increased bacterial infiltration in the intestinal epithelium; this results in the higher recruitment of macrophages and neutrophils in the inflammatory response [76]. Thus, it is observed that such an imbalance can lead to systemic inflammation with activation of the innate immune system. This occurs as some pathogenic bacteria, including *Salmonella typhimurium* and *C. difficile*, stimulate the innate immune system to produce pro-inflammatory cytokines such as IL-1, IL-6, and tumoral necrosis factor alpha (TNF-α) [73].

The pro-inflammatory cytokines, when in excess, can reflect in the fetal health, causing permanent damage, as is the case of IL-6 and Interleukin 17 (IL-17), since they harm the oligodendrocytes during brain development, which can cause permanent brain lesions due to the impairment of myelinization [77,78]. The combination of maternal chemokines and pro-inflammatory cytokines cross the placenta and can act in fetal brain development, stimulating inflammatory mediators that damage the brain, affecting its epigenetics, and leading to harmful effects on its plasticity, the migration of precursors, synapse formation and pruning, thus impairing its cognitive functions [78].

Nevertheless, a prospective cohort study observed that the increased maternal level of Interleukin 4 (IL-4), an anti-inflammatory cytokine, in the 28th week of pregnancy relates to a higher number of childbirths with signs of autism. In contrast, it was observed that the higher the concentrations of Interleukin 10 (IL-10) and anti-inflammatory agents, the lower the autism symptoms. This is justified since IL-4 is associated with the response of increased Th2 cells associated with producing antibodies and eliminating antigens in a normal situation. However, if this is maintained or occurs where the antigen is an autoantigen, it can prolong the inflammation [79].

It is known that diets rich in n-3 fatty acids lead to the production of anti-inflammatory metabolites [80]. In this scenario, the administration of fish oil is an often-used treatment in restoring the infiltration of pro-inflammatory cells and diminishing inflammation. Such results are attributed to the presence of DHA and EPA, which can restore the microbiota and normalize the inflammatory response [76]. These fatty acids can also cross the hematoencephalic barrier, acting during cognitive processes and playing essential roles in neurogenesis, neurotransmission, and protection against oxidative stress [81].

A study with female pups of Sprangue-Dawley rats observed that the administration of fish oil (corresponding to 18% of the diet during pregnancy and lactation), with 0.84 g/100 g of α-linolenic fatty acid and 49 g/100 g of the ratio DHA/EPA, was responsible for increasing the quantity of Bacteroidetes and controlling the balance of the immune response, thus serving to prevent inflammation [80]. Moreover, concerning the microbiota composition, the supplementation of a high dosage of n-3 PUFAs (1.0 g/kg/day) with 80% EPA/20% DHA led to an increase in *Actinobacteria* and a reduction in *Proteobacteria* [82].

Another work found similar results concerning the composition of the gut microorganisms of wild-type C57BL/6J male mice under a high-fat diet with linseed and fish oils. This diet, given over 16 weeks in wild-type C57BL/6J male mice, was responsible for increasing the concentrations of Bifidobacteriaceae and Bifidobacterium [81]. In contrast, consuming some fish with high mercury content does not demonstrate the same benefits in fetal neurodevelopment since this metal has a deleterious effect on fetal brain development. Thus, it is suggested that fish oil supplementation would be most suitable during pregnancy [83].

In the case of autistic children, the ratio between the phyla Bacteroidetes and Firmicutes is altered when compared with children without neurological alterations. When this imbalance occurs, the ratio of Bacteroidetes can increase, with pathogenic characteristics due to the presence of lipopolysaccharides (LPS) that have a detrimental effect on the immunological system of the host to cross the hematoencephalic barrier, increasing the concentration of mercury in the brain and diminishing the level of glutathione, an essential antioxidant for the detoxication of heavy metals [84].

It was also observed that the microbiota composition of the mother is similar to that found in her offspring, especially when fed with a high-fat diet [85]. A study with germ-free C57BL/6J mice receiving a Western-type diet for 10 weeks reported that the mother’s diet during pregnancy and lactation altered her composition of gut microorganisms and the neonate qualitatively [86]. Likewise, exposure to a diet rich in n-3 PUFAs during the gestational period molds the intestinal microbiota of the offspring and protects against metabolic alterations induced by HFD [87]. Although some studies have explored the relationship between dietetic supplementation with n-3 PUFAs and neuropsychiatric diseases, the impact on the microbiota, symptoms, and severity of patients with depressive disorder, ASD, or schizophrenia is still little understood [72].

Alterations in the gut microbiota composition are common during pregnancy, as well as other metabolic changes characteristic of this period [67,88]. For example, it is possible to observe the predominance of the content of the phylum Firmicutes during the first trimester, contrasting to the increase in the total content of the genus *Bifidobacteria* belonging to the phylum Actinomycetota and of the phylum Proteobacteria after this period. Moreover, bacteria of other organs, such as the vagina and oral cavity, also present changes concerning their composition during pregnancy due to hormonal and diet alterations [88].

Thus, it is possible to observe that changes in the composition of the gut microbiota, even if negligible, increase the susceptibility to the development of neurological disorders, such as ASD, and can aggravate the symptoms [25,66,86]. Such changes become more important when observed early, in the first two years of life, since this is a critical period for development and brain maturation, reaching 75% of its adult size at the end of the first 1000 days [46]. In a study with female mice fed a high-fat diet containing 60% fat for 8 weeks, it was observed that their offspring presented low sociability and interactions with other animals. Moreover, they presented repetitive behaviors and signs of anxiety, common characteristics in autistic individuals. Such behavioral alterations were attributed to the qualitative changes in the gut microbiota [85].

Short-chain fatty acids play a relevant role due to the decrease in butyrate, an example of a short-chain fatty acid whose reduction can lead to inflammation through mechanisms that include increased synthesis of nuclear factor kappa B (NF-kB) and absorption of lipopolysaccharides (LPS), which may increase the permeability of the blood–brain barrier, contributing to neuroinflammation [89]. In turn, the fatty acids n-3 PUFAs of food origin can provide adequate plasmatic and brain levels of EPA and DHA, which are substrates of lipoxygenases (LOXs) and cytochrome P450 (CYP450) and are constantly associated with a potent anti-inflammatory action, opposing either to the expression of pro-inflammatory cytokines, such as TNF-α, IL-6 and IL-1β, as to inflammatory stimuli induced by LPS [90].

Besides the maternal diet, other factors were found related to autism, such as gestational stress, the use of tobacco and alcoholic drinks, and breastfeeding practices, due to changes that occur in the oral and gut microbiota of the neonate, which are reflected directly in intestinal colonization in later years [21,91]. Such changes can influence the behavioral attitudes associated with autism [85]. Other effects of lipid intake in pregnancy concerning a higher or lower risk for autism should be better assessed.

## 6. Conclusions

Despite several difficulties in performing studies with the ASD population, most experimental and observational results reveal a potential correlation with adequate n-3 PUFA in the diet or supplements during the pregnancy, lactation, and pre-pregnancy phases, which presented lower levels of neurodevelopmental disorders and ASD development risk for their offspring. This correlation seems to be associated with less maternal intestinal dysbiosis, which corrects to the Firmicutes and Bacteroidetes ratio, consequently demonstrating the anti-inflammatory effects of n-3 PUFAs, which modulate the maternal–child microbiota quality and contribute to children’s myelination.

After this review, it is possible to perceive the strict number of studies emphasizing the approached theme; thus, additional studies are necessary to determine how dietetic fatty acids can modify the intestinal maternal microbial ecosystem and contribute to the development of ASD in their offspring.
